# Predictors of catheter-related bladder discomfort after gynaecological surgery

**DOI:** 10.1186/s12871-020-01018-6

**Published:** 2020-04-28

**Authors:** S. Y. Li, L. P. Song, Y. S. Ma, X. M. Lin

**Affiliations:** Department of Anesthesiology, West China Second Hospital of Sichuan University, Key Laboratory of Birth Defects and Related Diseases of Women and Children, Ministry of Education, No.20, Section 3, Renmin Nanlu, Chengdu, China

**Keywords:** Catheter-related bladder discomfort, CRBD, Predictive factors

## Abstract

**Background:**

Urinary catheterization is universally used during surgery, and the incidence of postoperative catheter-related bladder discomfort (CRBD) is very high during recovery. We conducted this study to identify the incidence and predictors of postoperative CRBD after gynaecological surgery in the post-anesthesia care unit (PACU).

**Methods:**

This was a prospective observational study. Patients undergoing gynaecological surgery under general anesthesia with intra-operative urinary catheterization were enrolled. We collected the clinical data, incidence and severity of CRBD, and postoperative pain for the patients. Predictive factors of CRBD were analysed by univariate and multivariate analysis.

**Results:**

A total of 407 patients were included in this study. The incidence of CRBD after gynaecological surgery was 64.6% (mild CRBD: 22.8%; moderate CRBD: 34.2%; and severe CRBD: 7.6%). Univariate analysis showed that age, type of surgery, type of laparoscopic surgery, additional analgesics, and postoperative pain were influencing factors for CRBD. Based on multivariate logistic regression analysis, age ≥ 50 years, uterus-related laparoscopic surgery, and lack of additional analgesics were independent predictors of moderate or severe CRBD.

**Conclusions:**

This observational study revealed that the incidence of CRBD after gynaecological surgery in PACU was very high. Age ≥ 50 years, uterus-related laparoscopic surgery, and lack of additional analgesics were independent predictors of CRBD.

**Trial registration:**

ChiCTR1800016390. Registered on 30 May 2018.

## Background

Urinary catheterization is widely used to avoid bladder retention, allow urine output measurement and blood volume assessment in patients during surgery. A catheter located in the bladder may cause discomfort postoperatively, and this is called catheter-related bladder discomfort (CRBD). With the extensive use of catheters, the incidence of CRBD has been rising, ranging from 47 to 90% postoperatively [1, 2]. The clinical manifestation of CRBD is similar to that of an overactive bladder (OAB), including urinary urgency, urinary frequency with or without urge incontinence, or discomfort in the supra-pubic region [3]. CRBD is so distressing that it can increase postoperative agitation and pain, reduce satisfaction of personal hospital stay, and even increase the workload of medical staff. Therefore, it is helpful to identify predictive factors for CRBD and to enable preventive measures in clinical practice.

The major two independent predictors of CRBD are male gender and a Foley catheter diameter greater than or equal to 18 Fr [1]. In addition, urinary catheter-related pain (UCRP) ≥ 4, obstetric and gynaecological surgeries, and age < 50 years are identified as postoperative risk factors for CRBD [4]. There is also a study showing that abdominal open surgery and a history of catheterization 3 months prior to the operation are independent predictors of CRBD after urological surgery [5].

Although obstetric and gynaecological surgeries have a higher incidence of CRBD, there is no study to date about the predictors of CRBD after gynaecological surgery. Therefore, we conducted this study to identify the incidence and predictors of postoperative CRBD after gynaecological surgery in the post-anesthesia care unit (PACU).

## Methods

This prospective observational study was approved by the China Ethics Committee of Registering Clinical Trials, and registered in the Chinese Clinical Trial Registry (ChiCTR1800016390). It was implemented in West China Second Hospital of Sichuan University from June to July 2018. The selection criteria included age ≥ 18 years, elective gynaecological operation which were not associated with intra-operative injury to the urinary tract or intestinal tract, requiring bladder catheterization. The exclusion criteria included patients with a history of OAB, bladder outflow obstruction, neurogenic bladder, preoperative urinary tract infection, or unable to communicate.

General anesthesia was implemented using a standardized approach in our hospital. Anesthesia was induced with midazolam, sufentanil, propofol, muscle relaxants, and maintained with sevoflurane or propofol. Lornoxicam and tramadol were common used as additional analgesia administered near the end of operation for postoperative pain according to anesthetists’ own habit without consideration of the effects on CRBD. Neostigmine and atropine were used to antagonize the residual effects of muscle relaxants. All patients received a 16-Fr Foley urinary catheter with 10 mL normal saline inflating the catheter balloon.

After tracheal extubation in the operating room, all patients were transferred to the PACU for further recovery. In the PACU, we collected clinical data for the patients, including age, sufentanil dosage, surgery duration time, type of surgery, type of laparoscopic surgery, occasion of catheterization (before anaesthesia or after anaesthesia), additional analgesics near the end of the operation, intraoperative atropine, and postoperative neostigmine and atropine. We classified gynaecological surgery into three types: laparoscopic surgery, open abdominal surgery, and cervical conization and pelvic reconstructive surgery. The laparoscopic surgery was classified as uterus-related laparoscopic surgery and non-uterine related laparoscopic in detail. We also evaluated the severity of CRBD and postoperative pain. Patients were instructed to differentiate CRBD from incisional or surgical pain.

The severity of CRBD was assessed as follows: none, did not report any CRBD even when asked; mild, revealed only on questioning; moderate, complained on their own without questioning but not accompanied with any behavioral response; severe, stated on their own and followed by behavioral responses such as strong verbal response, flailing limbs, or even try to pull out the urinary catheter. Postoperative pain was recorded as a visual analogue scale (VAS) score with 10 points.

Patients were divided into groups according to the incidence and severity of CRBD.

The incidence group was CRBD ≥1(mild, moderate and severe), while the severity group was CRBD≥2 (moderate and severe). Categorical variables were analyzed by the chi-square test and Fisher’s exact test. Multivariate logistic regression was used to assess predictors with *P* < 0.05 in univariate analysis. All the data were analyzed by using SPSS 17.0, and P < 0.05 was considered significant.

## Results

A total of 407 patients who underwent elective gynaecological surgery were included in this study. The incidence of CRBD was 64.6%, and the occurrence of moderate or severe CRBD was 41.8% in the PACU (Table 1). Patient characteristics are listed in Table 2. Univariate analysis showed age ≥ 50 years, cervical conization and pelvic surgery, uterus-related laparoscopic surgery, lack of additional analgesics, and VAS ≥ 4 to be predictive factors of CRBD (Table 3). In addition, multivariate logistic regression analysis showed that age ≥ 50 years and uterus-related laparoscopic surgery were independent predictors of the incidence of CRBD; and that age ≥ 50 years, uterus-related laparoscopic surgery, and lack of additional analgesics were independent predictors of moderate or severe CRBD (Table 4).

**Table 1:** Incidence and severity of CRBD after gynecological sugery in PACU. Data are expressed as number of patients (%)

CRBD	n (%)
NO	144 (33.4%)
Mild	93 (22.8%)
Moderate	139 (34.2%)
Severe	31 (7.6%)

**Table 2:** Patient characteristics. Incidence of CRBD: CRBD≥1, and severity CRBD: CRBD≥2

	CRBD≥1(*n* = 263)		CRBD< 1(*n* = 144)		CRBD≥2(*n* = 170)		CRBD< 2(*n* = 237)	
	Mean	SD	Mean	SD	Mean	SD	Mean	SD
Age	44.3	12,6	39.0	12.1	44.4	11.8	41.0	13.1
Height	159.9	28.5	157.9	9.9	158.3	5.0	159.8	30.7
Weight	58.1	8.5	57.4	12.2	58.4	8.6	57.5	10.8
Sufentanil dosage	22.4	6.5	22.7	7.1	22.3	6.2	22.7	7.1
Surgery duration time	122.6	68.8	125.4	84.2	122.3	65.2	124.5	80.7
Postoperative pain	3.4	1.5	2.8	1.8	3.6	1.4	2.9	1.8

**Table 3:** Univariate analysis for predictive factors of the incidence of CRBD (CRBD≥1) and the severity CRBD (CRBD≥2). Data are presented as number (%)

Variable	n	CRBD≥1 (incidence)	P	CRBD≥2 (severity)	P
Age					
≥ 50 y	100	78 (78.0)	0.001	51 (51.0)	0.031
<50 y	307	185 (60.3)		119 (38.8)	
Sufentanil dosage					
>20μg	180	121 (67.2)	0.328	78 (43.4)	0.569
≤ 20μg	227	142 (62.6)		92 (40.5)	
Surgery duration time					
>180 min	90	56 (62.2)	0.59	32 (35.6)	0.176
≤ 180 min	317	207 (65.3)		138 (43.5)	
Type of surgery:					
Laparoscopic surgery	278	175 (62.9)	0.009	115 (41.4)	0.032
Open abdominal surgery	80	47 (58.8)		27 (33.8)	
Cervical conization and pelvic reconstructive surgery	49	41 (83.7)		28 (57.1)	
Type of laparoscopic surgery:					
Uterus-related laparoscopic surgery	158	113 (71.5)	0.002	78 (49.4)	0.004
Non-uterine related laparoscopic surgery	121	66 (54.5)		40 (33.0)	
Occasion of catheterization:					
Before anaesthesia	68	39 (57.4)	0.170	22 (32.4)	0.085
After anaesthesia	339	224 (66.1)		148 (43.7)	
Additional analgesics:					
Yes	54	25 (46.3)	0.002	11 (20.4)	0. 001
No	353	238 (67.4)		159 (45.0)	
Intraoperative atropine:					
Yes	121	84 (69.4)	0.188	55 (45.5)	0.327
No	286	179 (62.6)		115 (40.2)	
Postoperative neostigmine and atropine:					
Yes	54	35 (64.8)	0.974	25 (46.3)	0.469
No	353	228 (63.6)		145 (41.1)	
Postoperative pain:					
VAS ≥ 4	139	100 (71.9)	0.026	70 (50.4)	0.011
VAS ≤ 3	268	163 (60.8)		100 (37.3)

**Table 4:** Multivariate logistic regression analysis for predictive factors of the incidence of CRBD (CRBD≥1) and the severity of CRBD (CRBD≥2)

	CRBD≥1(incidence)			CRBD≥2(severity)		
	Odd ratio	95% CI	P	Odd ratio	95% CI	P
Age	3.203	[1.6, 6.6]	0.002	2.106	[1.2, 3.8]	0.013
≥ 50 y						
<50 y						
Sufentanil dosage	1.475	[0.9, 2.4]	0.111	1.416	[0.9, 2.2]	0.133
>20μg						
≤ 20μg						
Surgery duration time	0.783	[0.4, 1.4]	0.403	0.655	[0.4, 1.2]	0.141
>180 min						
≤ 180 min						
Type of surgery:	2.297	[0.7, 7.2]	0.153	1.898	[0.8, 4.4]	0.132
Laparoscopic surgery						
Open abdominal surgery						
Cervical conization and pelvic surgery						
Type of laparoscopic surgery:	1.899	[1.1, 3.2]	0.017	1.863	[1.1, 3.1]	0.019
Uterus-related surgery						
Non-uterine related surgery						
Occasion of catheterization:	0.633	[0.4, 1.1]	0.125	0.614	[0.3, 1.1]	0.109
Before anaesthesia						
After anaesthesia						
Additional analgesics:	0.509	[0.2, 1.1]	0.072	0.408	[0.2, 0.9]	0.032
Yes						
No						
Intraoperative atropine:	1.414	[0.9, 2.3]	0.152	1.370	[0.9, 2.2]	0.172
Yes						
No						
Postoperative neostigmine and atropine:	0.767	[0.9, 2.3]	1.098	1.347	[0.7, 2.5]	0.331
Yes						
No						
Postoperative pain:	1.742	[1.0, 3.2]	0.066	1.517	[0.9, 2.6]	0.133
VAS ≥ 4						
VAS ≤ 3						

## Discussion

According to this observational study, the incidence of CRBD after gynaecological surgery was 64.6%, and the occurrence of moderate or severe CRBD was 41.8%. Age ≥ 50 years, uterus-related laparoscopic surgery, and lack of additional analgesics might be the independent predictive factors of CRBD after gynaecological surgery.

Age ≥ 50 year was associated with a higher incidence and severity of CRBD in our study, in contrast to the results of Lim’s study [4]. In gynaecological surgery, more malignant lesions and hysterectomy-related surgery in older people have been reported, with more benign lesions and non-hysterectomy-related surgery in younger individuals. This was consistent with our finding that hysterectomy-related laparoscopic surgery was correlated with a higher incidence and severity of CRBD than was non-hysterectomy-related laparoscopic surgery. Because the uterus is adjacent the bladder, placement of the uterine manipulator is likely to stimulate the bladder during hysterectomy-related laparoscopic surgery. In addition, postoperative loss of peripheral tissue support can easily induce bladder paralysis. Furthermore, postoperative surgical-site pain might aggravate CRBD.

Cervical conization and pelvic reconstructive surgery resulted in a higher incidence and severity of CRBD. This might be related to the surgical procedures, whereby pulling the urethra to expose the vagina and cervix might stimulate the urethra intra-operatively, and the oil gauze/ iodophor gauze filling the cervix/vagina may compress the urethra postoperatively. Our study also showed that additional analgesics administered near the end of the operation and postoperative pain VAS ≤ 3 were associated with a lower incidence and severity of CRBD. Studies have reported that tramadol and non-steroid anti-inflammatory drugs are effective for managing CRBD [6, 7]. Moreover, patients might confuse surgery-related pain with urinary catheter-related pain.

The mechanism of CRBD is due to the disordered bladder contraction mediated by muscarinic receptors, especially subtype M3 receptors [8]. Various antimuscarinic agents, such as tolterodine, oxybutynin, butylscopolamine, ketamine, tramadol, and dexmedetomidine, have been employed to reduce CRBD with varying degrees of success [9–15]. Nonetheless, these drugs also have some adverse effects, such as dry mouth, sedation, nausea, and vomiting. Thus, we should weigh the advantages and disadvantages of CRBD and adopt a multi-mode comprehensive prevention and control method to manage it. These methods include lubricating oil, local anesthetics, psychological intervention, drug prevention (used for high-risk patients: male gender, urological surgery, or obstetric and gynaecological surgery), and medical treatment (needed for moderate or severe CRBD postoperatively). Previous report suggested that sevoflurane with antimuscarinic effect decrease the incidence of CRBD [16]. As our hospital conventionally used sevoflurane inhalation to maintain anesthesia, the including patients in this study were all used sevoflurane.

This study has some limitations. First, we only evaluated the incidence and severity of CRBD in the PACU, but we did not perform further evaluation in the ward, some issues with CRBD might occur after PACU and on the ward. Besides, the size of urinary catheter is a risk factor of CRBD. In this study, 16 Fr. Urinary Catheter was used in all patients. The high incidence of CRBD in this investigation may be related to the big-sized urinary catheter. Moreover, we did not observe the post-operative urinary tract infection. There was a certain imbalance in the primary data, such as type of surgery, additional analgesics, and occasion of catheterization. For example, there was more laparoscopic surgery and less cervical conization and pelvic reconstructive surgery in our hospital. The patients used additional analgesics and catheterized before anaesthesia were also less.

## Conclusion

This observational study revealed that the incidence of CRBD after gynaecological surgery in PACU was very high. Age ≥ 50 years, uterus-related laparoscopic surgery, and lack of additional analgesics were independent predictors of CRBD.

## Data Availability

The datasets are not publicly available, but available from the corresponding author on reasonable request.

## Abbreviations

CRBD: catheter-related bladder discomfort
PACU: post-anesthesia care unit
OAB: overactive bladder
UCRP: urinary catheter-related pain
VAS: visual analogue scale
